# Patients’ high acceptability of a future therapeutic HIV vaccine in France: a French paradox?

**DOI:** 10.1186/s12879-019-4056-6

**Published:** 2019-05-09

**Authors:** Svetlane Dimi, David Zucman, Olivier Chassany, Christophe Lalanne, Thierry Prazuck, Emmanuel Mortier, Catherine Majerholc, Isabelle Aubin-Auger, Pierre Verger, Martin Duracinsky

**Affiliations:** 1 Department of Internal Medicine, Réseau Ville Hôpital Val de Seine, Foch Hospital, Suresnes, France; 2 EA 7334 REMES, Patient-Centered Outcomes Research, University Paris-Diderot, Sorbonne Paris Cité, Paris, France; 3 Clinical Research Unit in Health Economics (URC-ECO), Fernand Widal Hospital, AP-HP, Paris, France; 4 Department of Infectious Diseases, Regional Hospital Orléans, Orléans, France; 5 Department of Internal Medicine, Louis Mourier Hospital, Colombes, France; 6 EA 7334 REMES, University Paris-Diderot, Sorbonne Paris Cité, Paris, France; 7 Observatoire régional de la santé Paca, Marseille, France; 8 AMU-UMR912 SESSTIM-IRD, Marseille France, Marseille, France; 9 Department of Internal Medicine & Clinical Immunology, Bicetre Hospital, AP-HP, Kremlin-Bicetre, Paris, France

**Keywords:** HIV, Therapeutic vaccine, Acceptability, Acceptance, Confidence

## Abstract

**Background:**

France is the European country with the lowest level of confidence in vaccines. Measurement of patients’ acceptability towards a future therapeutic HIV vaccine is critically important. Thus, the aim of this study was to evaluate patients’ acceptability of a future therapeutic HIV vaccine in a representative cohort of French patients living with HIV-AIDS (PLWHs).

**Methods:**

This multicentre study used quantitative and qualitative methods to assess PLWHs’ opinions and their potential acceptance of a future therapeutic HIV vaccine. Cross-sectional study on 220 HIV-1 infected outpatients, aged 18–75 years.

**Results:**

The participants’ characteristics were similar to those of the overall French PLWH population. Responses from the questionnaires showed high indices of acceptance: the mean score for acceptability on the Visual Analog Scale VAS was 8.4 of 10, and 92% of patients agreed to be vaccinated if a therapeutic vaccine became available. Acceptability depended on the expected characteristics of the vaccine, notably the duration of its effectiveness: 44% of participants expected it to be effective for life. This acceptance was not associated with socio-demographic, clinical (mode of contamination, duration of disease), quality of life, or illness-perception parameters. Acceptability was also strongly correlated with confidence in the treating physician.

**Conclusion:**

The PLWHs within our cohort had high indices of acceptance to a future therapeutic HIV vaccine.

**Trial registration:**

This study was retroactively registered on ClinicalTrials.gov with ID: NCT02077101 in February 21, 2014.

## Background

In developed countries, where effective antiretroviral treatment (ART) is available, people living with HIV (PLWH) have acquired a long-term positive prognoses [1]. HIV infection is now a chronic disease but there is a patient demand for a cure. There is currently intense research into the development of a HIV therapeutic vaccine to obtain sustained ART-free HIV remission. It is expected that, in the future, PLWH will benefit from such vaccines [2–4].

There is also intense research on an HIV vaccine to prevent disease transmission and possibly eradicate the pandemic [5–7]. HIV-preventive and HIV-therapeutic vaccine research benefit from each other’s results and improve our understanding of the mechanisms of immune protection against HIV. Six efficacy trials that have been conducted to date, and only one, the RV144 Thai trial of ALVAC/gp120, showed a modest protective efficacy [8]. But these results has provided important lessons for future strategies towards a cure for HIV [9].

The WHO Strategic Advisory Group of Experts (SAGE) on Immunization has recognized that reservations about vaccines (vaccine hesitancy) are a growing global problem [10, 11].

Anticipation of the “acceptability” of vaccines by the public has become an increasingly important factor in the development of new vaccines [12].

Many studies have shown the crucial role of General Practitioners (GPs) in promoting and proposing preventive vaccination [13]. GPs are the gatekeepers of the French health system, and are usually consulted by their patients regarding vaccination issues [13–18]. France is the European country with a highest vaccine hesitancy [19]. In the French general population, refusal of vaccination has become frequent, particularly for the hepatitis B and for the influenza vaccine especially as many internet sites report frightening consequences of vaccination. In 2009, 24% of French GPs had a “vaccination hesitancy” profile [20–22] and remains high.

Attitudes to vaccination are a continuum ranging from total acceptance to complete refusal [23].

In the general population, the determinants of acceptance of HIV-preventive vaccination have been studied [24–26]. Newman et al. in 2010, reported in a meta-analysis a high acceptability of HIV preventive vaccination, which was correlated with vaccine effectiveness and duration of protection. Barriers were correlated with fear of side effects and of syringes [24].

There is only one publication on the willingness to participate in HIV Therapeutic Vaccine Trials among HIV-Infected Patients on ART in China. This study showed high acceptability [27].

In France, due to the complex attitudes of the general population, we considered important to study if the future HIV therapeutic vaccines will be acceptable when they will become available.

To this effect, the Representations and Acceptability of a Therapeutic HIV Vaccine (RAVVIH) study was designed to explore the perceptions of therapeutic HIV vaccines in a cohort of HIV outpatients.

## Methods

The objective of this study was to assess the acceptability of a future HIV therapeutic vaccine in HIV-positive outpatients aged 18–75 years. Univariate and multivariate analyses were conducted to find the factors correlated to HIV vaccine acceptability.

### Study design

The RAVVIH study was a prospective cross-sectional conducted in three hospitals. The three infectious disease departments that participated in this study were selected to be representative of French AIDS-care centers. One is a private tertiary hospital (Hôpital Foch), Hôpital Louis Mourier is a University hospital in the Paris suburbs, and CHR d’Orleans is a public provincial hospital.

### Study participants

Between December 2013 and May 2014, consecutive outpatients were solicited during their biannual HIV visit to their HIV physician. Eligibility criteria were: being aged between 18 and 75 years, being infected with HIV-1 and having French medical insurance coverage. Patients who did not speak French language were excluded. Questionnaire administration and interviews took place at the patient’s care hospital.

## Methods

### Quantitative study

Patients completed three self-administered questionnaires: two that had previously been validated (Brief IPQ-R [28] and PROQOL-HIV [29]) and a specific questionnaire on vaccination: the RAVVIH questionnaire; they also scored a Visual Analog Scale (VAS) of acceptability.

The Brief Illness Perception Questionnaire-Revised (Brief IPQ-R) is a nine-item scale designed to rapidly assess the cognitive and emotional representations of illness. The Brief IPQ-R uses a single-item approach to assess perception on a 0–10 response scale. It comprises items on cognitive perceptions of illness: consequences, timeline, personal control, treatment control and identity. This questionnaire explores patients’ own beliefs about their condition [30].

The Patient-Reported Outcomes Quality Of Life specific HIV instrument (PROQOL-HIV) comprises 43 items, dealing with eight themes that dominate the experiences of HIV patients living in the ART era: General health perceptions, social relationships, emotions, energy/fatigue, sleep, cognitive functioning, physical and daily activities, coping, future and treatment’s impact. It was developed simultaneously across nine countries, in accordance with rigorous international standards [31, 32]. Scores for each dimension range from 0 to 100 (100 = best QoL). A four-dimension summary scoring scheme was recently proposed [33] which has been used in our study: physical health and symptoms (PHS), health concerns and mental distress (HCMD), social and intimate relationships (REL), and treatment-related impact (TRT).

The RAVVIH questionnaire includes 50 items regarding factors identified in several preventive acceptance studies. These items were selected by the RAVVIH study group (DZ, SD, MD) and tested by five PLWHs. It covers three main themes on vaccination: knowledge, representation and acceptability. Among these 50 items, 28 are 5-point Likert-type questions ranging from 1 (strongly disagrees) to 5 (strongly agrees), the others requiring dichotomous answers.

The Visual Analogue Scale (VAS) of acceptability is a horizontal analogue scale graduated from 0 (“Whatever my situation, I will never accept a therapeutic vaccine”) to 10 (“I see no problem in using a therapeutic vaccine as soon as one becomes available”), on which the patient expresses his/her level of agreement with the proposal using a check mark.

### Qualitative study

A review of the literature was performed to identify important HIV-vaccine acceptance determinants: doctor confidence, knowledge, perceptions of illness, secrecy, quality of life (Qol). These determinants were used as the main topics in the interview guide, 20 patients who did not participate in the quantitative study were interviewed. Semi-structured face−to−face interviews were performed by a trained psychologist (IP) until data saturation was obtained [33]. The interviews, of 45–60 min duration, were recorded and transcribed verbatim. A triangulation analysis was performed by two experienced researchers (IA and LB). Open coding was performed within a framework predefined by the themes in the interview guide. A common list was used and enriched for further analysis, which was carried out manually.

### Study outcomes

The primary outcome was the therapeutic HIV-vaccine acceptability on the Visual Analog Scale (VAS). Secondary outcomes were factors associated with vaccine acceptability according to the questionnaires, answers to the Brief Illness Perception Scale (Brief IPQ-R), and to the PROQOL-HIV questionnaire.

### Statistical analyses

A preliminary power analysis indicated that a total of 200 participants was required to estimate a mean score VAS with a margin of error (half width of a 95% confidence interval) less than 0.5 point assuming a standard deviation of 1 point. The distribution of acceptability scores was summarized using means, medians, standard deviations and interquartile ranges for continuous variables and counts and proportions for categorical data. Two-group comparisons were performed using the Mann–Whitney test in cases of continuous outcomes and Pearson’s chi-square test in cases of categorical variables. Multivariate analysis was also used to explore responses to the 50 items of the RAVVIH questionnaire. Pearson’s correlations were used to reduce the 28 Likert-type questions to a subset of variables that correlate above 0.25 to the therapeutic HIV-vaccine acceptability VAS. Principal-component analysis was carried out on this subset of 28 variables (the Likert-type responses being considered as continuous). Only complete cases with no missing responses were included for this analysis. All statistical tests were two-tailed with a significance level at 5%. The R software (The R Foundation, Vienna, Austria) was used for all statistical analyses.

### Ethics

All subjects provided their written consent prior to the study. Questionnaires and interviews were fully anonymous. A favorable ethical opinion was obtained from the relevant French Ethics Committee (IDRCB 2013-A01344–41).

### Clinical trial registration

This study was registered retroactively on ClinicalTrials.gov with ID: NCT0207710.

## Results

On the global sample (qualitative and quantitative) *N* = 220, there were 5 missing data on sex.

Table 1 shows the clinical characteristics of the 215 (mean age ± SD: 48 ± 12).

**Table 1:** Clinical characteristics of patients *n*=215

	Female *N* = 64	Male *N* = 151	Total *N* = 215
Age [years]	44 (11)	50 (12)	48 (12)
Disease duration [years]	13 (8.9)	15 (8.9)	14 (8.9)
HIV infection transmission risk group			
Heterosexuals	89% (54)	27% (41)	45% (95)
MSM	0% (0)	59% (88)	42% (88)
Injecting drug users	8% (5)	11% (16)	10% (21)
Others	3% (2)	3% (5)	3% (7)
Duration of ART [years]	10 (7.3)	11 (7.0)	11 (7.0)
CDC clinical stage			
A	60% (35)	66% (80)	64% (115)
B	12% (7)	7% (9)	9% (16)
C	28% (16)	26% (31)	26% (47)
HIV viral load below detection limit	89% (56)	93% (138)	92% (194)
CD4 [cell count/mm]	631(286)	672 (296)	661 (286)
Co-morbidities			
Chronic hepatitis B (AgHbs+)	8% (5)	3% (5)	5% (10)
Hepatitis C (positive HCV serology)	12% (8)	15% (22)	14% (30)
HBV + HCV	2% (1)	1% (2)	1% (3)
Treated Diabetes	3% (2)	9% (13)	7% (15)
Treated Hypertension	20% (13)	30% (45)	27% (58)
Cancer	6% (4)	9% (13)	8% (17)
Vaccination coverage			
HBV vaccination:			
up to date	64% (38)	80% (116)	75% (154)
not up to date	20% (12)	9% (13)	12% (25)
don’t know	15% (9)	11% (16)	12% (25)
Diphtheria-tetanus-inactivated poliovirus vaccination dTp:			
up to date	67% (43)	75% (113)	73% (156)
not up to date	14% (9)	14% (21)	14% (30)
don’t know	19% (12)	11% (16)	13% (28)
Pneumococcal vaccination:			
updated	16% (10)	15% (22)	15% (32)
not up to date	62% (40)	70% (101)	67% (141)
don’t know	22% (14)	15% (22)	17% (36)
Influenza vaccination:			
up to date	19% (12)	23% (32)	21% (44)
not up to date	72% (46)	69% (98)	70% (144)
don’t know	9% (6)	8% (12)	9% (18)

Numerical variables are summarized using mean (SD); categorical variables are summarized using proportions (counts); MSM, men who have sex with men

70% (*N* = 151) were men, 59% of them (*N* = 88) being men who have sex with men; 21 patients (10%) were intravenous drug users.

26% of patients were at the CDC clinical stage C, but did not have any associated opportunistic infection since several years before the study.

Almost all patients (96%) were taking ART and 92% had an undetectable viral load.

### Quantitative study

#### Social patient characteristics

Majority of the sample had french nationality (80%), 58% completed high school and 88% had a current job.

#### VAS of therapeutic vaccine acceptability

The VAS score for the acceptability of a future therapeutic vaccine was (mean ± SD) 8.4 ± 2.2. Nearly half of the participants (46%) gave a score of 10. Those who reported in the RAVVIH questionnaire that they would accept therapeutic vaccination in the future had an average score of 8.9 ± 1.5 whereas those who were negative (“no” and “don’t know”) had a mean score of 5.0 ± 2.7 (Wilcoxon test, *p* < 0.001).

#### RAVVIH questionnaire

Sixty− 7 % of all patients considered their recommended vaccine coverage to be complete. Only 18% reported a history of side−effects from their last vaccine: mainly local pain, redness and swelling.

In the patients’ medical files, we found that immunization coverage for hepatitis B, diphtheria, tetanus and poliomyelitis was high at 75%. Immunization coverage was much lower for influenza (15%) and pneumococcus (21%).

The willingness to receive a therapeutic HIV vaccine was high (91%) if the referent HIV clinician recommended it, but 71% of participants feared possible side−effects. The mode of administration (syringe) was not a barrier for these participants (86%).

Willingness to participate in a therapeutic HIV-vaccine clinical trial was high (74%); only 9% of patients gave a negative response, and 17% had no opinion.

Patients would consider stopping ongoing ART if the efficacy of the therapeutic vaccine on HIV viral load was demonstrated to be between 85 and 100%.

Forty− 4 % of patients contended that lifelong cessation of ART was the only acceptable endpoint. Three to six months without taking ART was considered a satisfactory therapeutic goal by 40% of patients and 9 to 12 months was preferred by a minority (12%).

The more frequently expected benefits were stopping the treatment burden of daily pills (93%) and not to be a risk for HIV transmission to partner or children (84%).

Most participants agreed to be vaccinated if a therapeutic vaccine became available (92%). Thirty percent (57 patients) thought that *“doctors do not tell all the truth about vaccines”*.

Detailed results are provided in Table 2, which also shows those questionnaire Likert−type scales that were correlated to the acceptability VAS.

**Table 2:** Answers to the Likert scales of the RAVVIH questionnaire

Question	N	Strongly agree	Agree	Un-certain	Disagree	Strongly disagree	VAS correlation
*My treating general practitionner understands my disease*	196	128 (65.3)	40 (20.4)	20 (10.2)	4 (2.0)	4 (2.0)	0.001
*Generally we can trust the doctors*	198	103 (52.0)	80 (40.4)	11 (5.6)	4 (2.0)	–	0.174
*Doctors don’t tell us everything about vaccines*	196	15 (7.7)	42 (21.4)	51 (26.0)	61 (31.1)	27 (13.8)	0.035
*I have a good general knowledge on vaccination*	196	19 (9.7)	86 (43.9)	51 (26.0)	26 (13.3)	14 (7.1)	0.153
*I think vaccines are effective*	195	106 (54.4)	79 (40.5)	9 (4.6)	1 (0.5)	–	0.356^a^
*For vaccines to be effective several doses are important*	200	169 (84.5)	26 (3.0)	4 (2.0)	–	1 (0.5)	0.241
*For vaccines, I do not trust health public authorities*	193	16 (8.3)	43 (22.3)	65 (33.7)	51 (26.4)	18 (9.3)	0.177
*Health public authorities tend to hide informations*	193	25 (13.0)	54 (28.0)	62 (32.1)	41 (21.2)	11 (5.7)	0.122
*I do not trust pharmaceutical companies*	196	20 (10.2)	63 (32.1)	60 (30.6)	38 (19.4)	15 (7.7)	0.226
*One day, I believe they will find a vaccine to cure the HIV virus*	199	130 (65.3)	60 (30.2)	9 (4.5)	–	–	0.275^a^
*I understand what is a HIV therapeutic vaccine*	197	115 (58.4)	63 (32.0)	17 (8.6)	2 (1.0)	–	0.101
*I will agree if my doctor recommends it to me*	195	134 (68.7)	46 (23.6)	7 (3.6)	5 (2.6)	3 (1.5)	0.500^a^
*I prefer to stay on ART it is safer because I already know it*	174	21 (12.1)	32 (18.4)	51 (29.3)	42 (24.1)	28 (16.1)	0.403^a^
*If an effective therapeutic vaccine against HIV was available my sexual life would change*	200	60 (30.0)	35 (17.5)	49 (24.5)	36 (18.0)	20 (10.0)	0.046
*If an effective therapeutic vaccine against HIV was available I would have more sexual partners*	198	8 (4.0)	13 (6.6)	37 (18.7)	60 (30.3)	80 (40.4)	0.013
*If an effective therapeutic vaccine against HIV was available I would be less likely to use condoms*	198	26 (13.1)	26 (13.1)	27 (13.6)	56 (28.3)	63 (31.8)	0.005
*If an effective therapeutic vaccine against HIV was available I would have more satisfaction in your sexual life?*	196	49 (25.0)	40 (20.4)	49 (25.0)	34 (17.3)	24 (12.2)	0.142
*I am worried by the possible side effects (of an HIV therapeutic vaccine)*	198	58 (29.3)	82 (41.4)	32 (16.2)	19 (9.6)	7 (3.5)	0.321^a^
*As the vaccine is administered with a needle this would be an obstacle for me*	197	5 (2.5)	2 (1.0)	21 (10.7)	42 (21.3)	127 (64.5)	0.166
*I am worried because we do not still know the actual long-term efficiency of this vaccine*	198	40 (20.2)	78 (39.4)	43 (21.7)	21 (10.6)	16 (8.1)	0.427^a^
*I am worried that the vaccine can make me even sicker*	197	29 (14.7)	34 (17.3)	61 (31.0)	45 (22.8)	28 (14.2)	0.316^a^
*It worries me to know that a part of the vaccine is made with viral DNA*	197	18 (9.1)	20 (10.2)	69 (35.0)	45 (22.8)	45 (22.8)	0.294^a^
*I am worried because with the vaccine I cannot control what I take compared with the daily taking of antiretrovirals*	196	16 (8.2)	30 (15.3)	51 (26.0)	45 (23.0)	54 (27.6)	0.387^a^
*I expect a therapeutic HIV vaccine to improve my social life*	197	94 (47.7)	45 (22.8)	31 (15.7)	13 (6.6)	14 (7.1)	0.065
*I expect a therapeutic vaccine to allow me not to contaminate my close family/friends*	198	130 (65.7)	37 (18.7)	17 (8.6)	8 (4.0)	6 (3.0)	0.040
*I expect a therapeutic HIV vaccine to facilitate my professional life*	191	63 (33.0)	35 (18.3)	53 (27.7)	20 (10.5)	20 (10.5)	0.051
*I expect a therapeutic HIV vaccine to improve my sentimental life*	198	86 (43.4)	50 (25.3)	39 (19.7)	14 (7.1)	9 (4.5)	0.024
*I expect a therapeutic HIV vaccine to improve the daily constraints of taking treatement*	197	137 (69.5)	47 (23.9)	9 (4.6)	1 (0.5)	3 (1.5)	0.253^a^

Counts (percentage)

^a^significanly correlated to the VAS acceptability score

#### Evaluation of disease burden with the brief IPQ-R questionnaire

Participants were worried by the duration of their disease *(How long do you think your illness will continue?)* and by the impact of their symptoms *(How much do you experience symptoms from your illness?)*(mean ± SD: 2.1 ± 2.6). Answers were more positive concerning their antiretroviral treatment, and their understanding of their disease.

In general, PLWHs scored high on ‘treatment control’ (9.1 ± 1.6) (*How much do you think your treatment can help your illness?)* and ‘concern’ (8.1 ± 2.5) (*How concerned are you about your illness?).*

#### Evaluation of patients’ Qol with the PROQOL-HIV questionnaire

Except for the HCMD dimension, average scores were superior to 70 (on a 100 points scale), and 50% of the participants scored between 60 and 90 points, indicating that their Qol was intermediate to good. The lower HCMD score shows participants to be more bothered by stigma and sexual issues. The quality of their social relationships was considered satisfactory.

#### Multivariate analyses

Among the 28 Likert-type questions in the RAVVIH questionnaire, nine were correlated (Pearson’s r > 0.25) to VAS scores (Table 2).

**Fig. 1:**
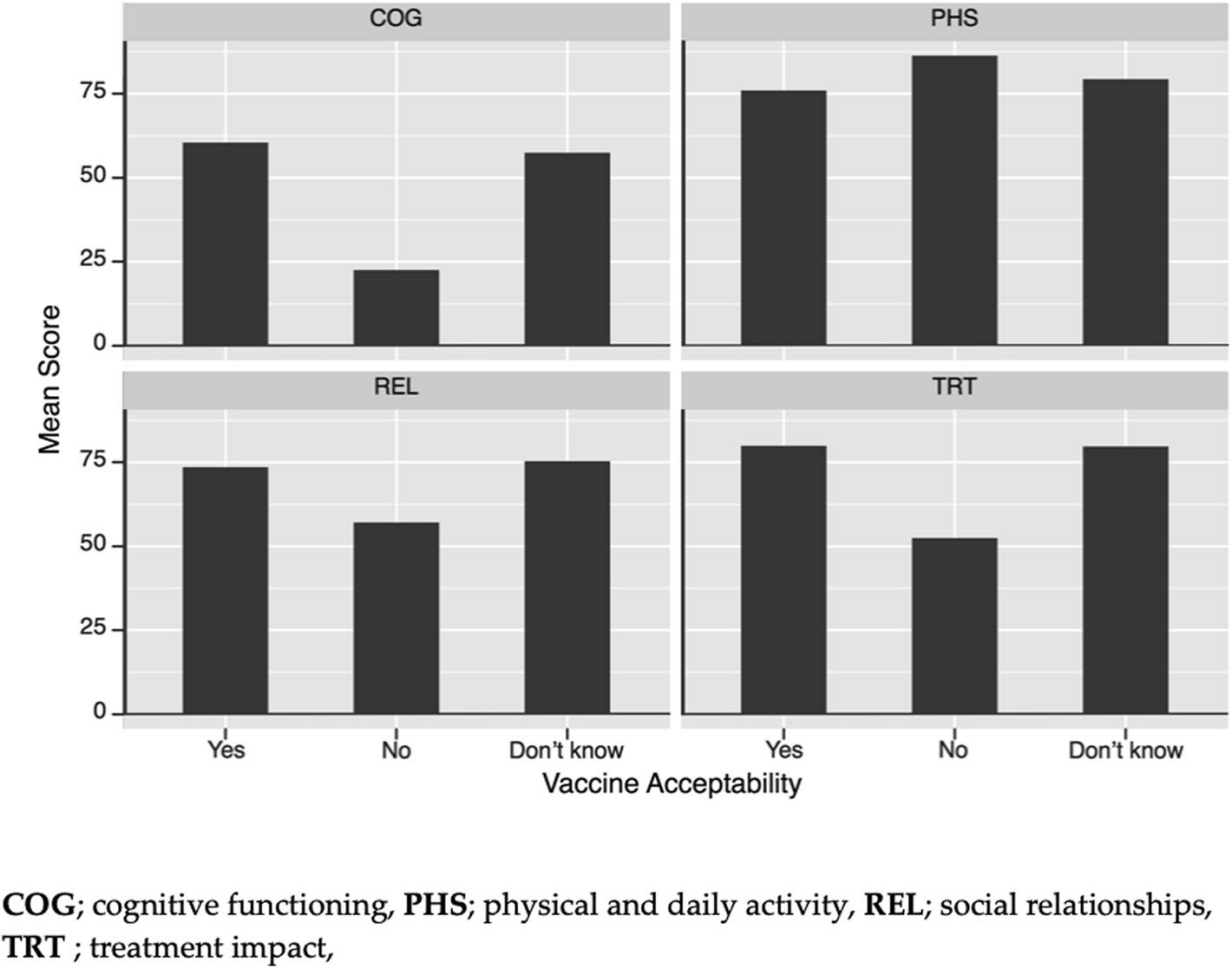
Acceptability and quality of life

A principal component analysis (PCA) on 149 participants with no missing responses gave a primary dimension that accounted for 35% of total variance, while the second dimension accounted for 13% of variance. The first dimension (Fig. 1) is clearly bidimensional opposing vaccine efficacy and recommendation by GP to control, DNA, ART side−effects, disease worsening and disease duration. The second dimension is one−dimensional and lumps together recommendations by GPs, ART constraints, vaccine development and side−effects. Individual factor scores from the first dimension of PCA were significantly correlated to the VAS on acceptability of a future therapeutic vaccine (Pearson *r* = 0.59, 95% CI [0.47;0.68], *P* < 0.005).

With respect to the IPQ-R, there is little difference between the total participants’ score according to their response on the acceptability of the therapeutic vaccine: “Yes”, 33.1 ± 10.6; “No”, 34.0; “Do not know”, 34.8 ± 9.6. By grouping the responses “No” and “Do not know”, there was no significant difference between the two groups of respondents (Wilcoxon test, *p* = 0.721).

The quality of life scores for each of the 4 dimensions of the PROQOL-HIV questionnaire according to the response on the acceptability of the therapeutic vaccine are summarized in Fig. 1. For the only patient who answered “No”, it is noteworthy that with the exception of the PHS dimension, the quality of life is greatly worse compared to other patients, especially on the COG dimension (22.5 vs 60.5 ± 23.3). The quality of life scores of patients who answered “Do not know” do not differ significantly from those who answered “yes”.

It should be noted that the total IPQ-R score remains well correlated with PROQOL-HIV dimension scores: PHS, *r* = − 0.533, *p* < 0.001; COG, *r* = − 0.525, *p* < 0.001; REL, *r* = − 0.588, *p* < 0.001; TRT, *r* = − 0.387, *p* < 0.001.

No difference of the acceptability of the future vaccine was found related to sociodemographic characteristics: gender (Pearson chi-2, *p* = 0.487, age (Welch t test, *p* = 0.521), level of education (undergraduate vs graduate, *p* = 0.688), or the socioprofessional status (stable vs. precarious employment *p* = 0.916). Similarly, year of HIV diagnosis, mode of transmission and ART duration did not influence the acceptability.

**Fig. 2:**
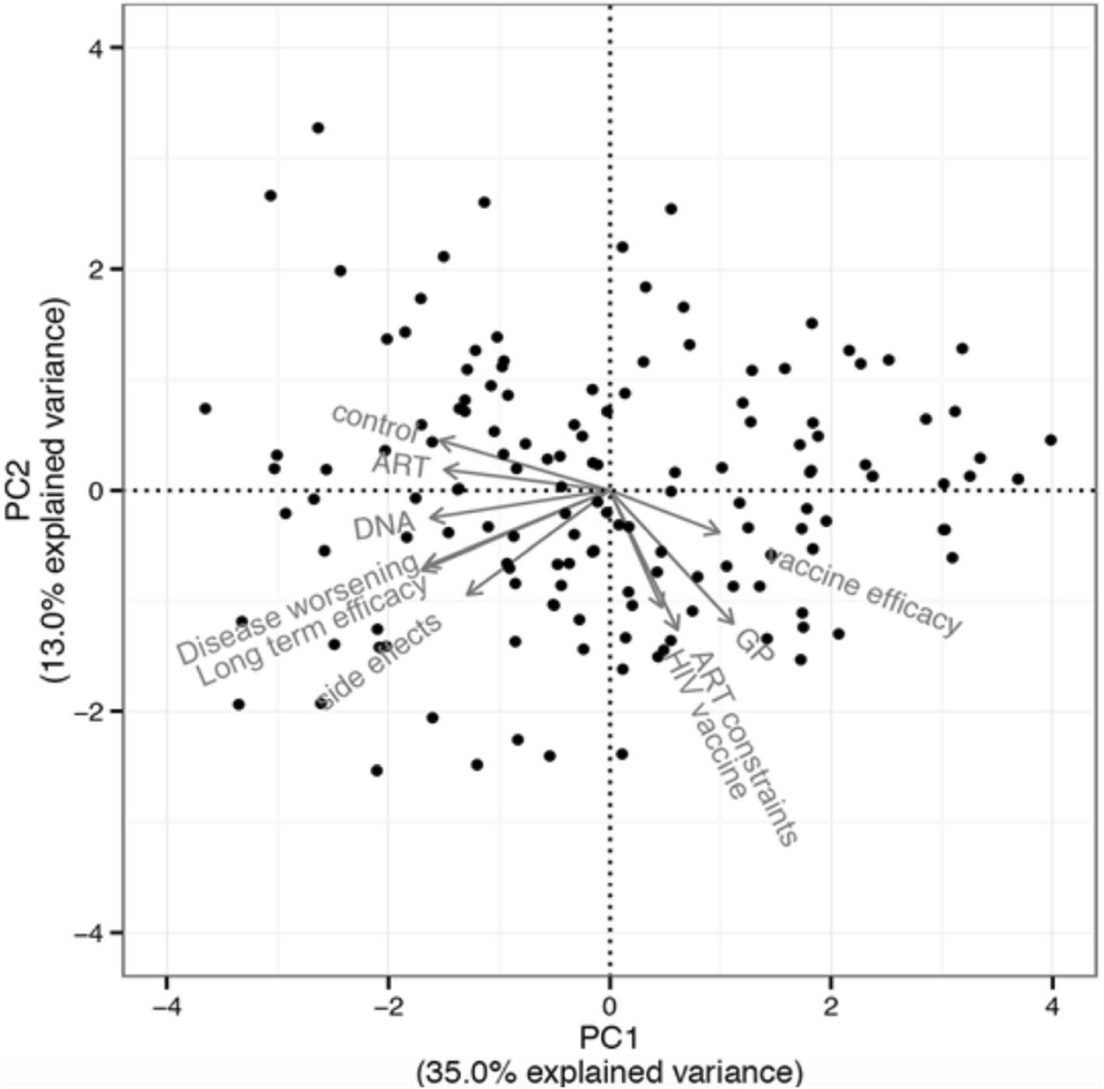
Multivariate analysis of the HIV vaccine acceptability. The angle formed by two vectors is proportional to the correlation between the two variables. Thus, the smaller the angle, the more the variables are correlated with one another; two vectors whose angle is approximately 180 ° indicates a strong negative correlation; finally, two vectors whose angle is 90 ° reflects and absence of correlation between the two variables associated with these vectors

Acceptability is presented considering different concepts emerging from factorial analysis. Some arrows are overlapping like ART constrains and HIV vaccine as well as long term efficiency and disease worsening. There is no correlation between the acceptability of the future HIV vaccine and the patients quality of life (Fig. 2).

### Qualitative analyses

The mean age of 20 participants was 46 years [range: 23–66], 13 were male and 14 had a CDC stage A. All patients were on ART and 95% had an undetectable viral load (Table 3).

i) Doctor–Patient relationship

**Table 3:** Characteristics of the 20 interviewees

Patient	Gender M/F	Duration of HIV diagnosis	CDC	ART	ART regimen
1	F	17	A	RAL/LPV	BID
2	M	23	A	TDF/FTC/DRV/r	OD
3	M	18	C	TDF/FTC/EFV	OD
4	M	32	A	TDF/FTC/RAL	BID
5	M	9	C	TDF/FTC/EFV	OD
6	M	6	A	TDF/FTC/RPV	OD
7	F	35	A	TDF/FTC/RAL	OD
8	M	32	C	TDF/FTC/DRV/r	OD
9	M	25	A	TDF/FTC	OD
10	M	33	A	ABC/3TC/EFV	OD
11	M	14	A	TDF/FTC/ETR	OD
12	M	7	A	TDF/FTC/RPV	OD
13	F	33	A	TDF/FTC/DRV/r	OD
14	F	18	C	TDF/FTC/LPV/r	BID
15	M	34	B	ABC/3TC/EFV	OD
16	M	32	A	TDF/FTC/DRV/r	BID
17	M	33	C	TDF/FTC/DRV/r	BID
18	F	22	A	ABC/3TC/ETV	OD
19	F	9	A	DRV/r	OD
20	F	7	A	TDF/FTC/EFV	OD

*Abbreviations: ART* active antiretroviral therapy, *3TC* lamivudine, *FTC* emtricitabine, *ABC* abacavir, *TDF* tenofovir, *ETV* etravirine, *RPV* rilpivirine, *EFV* efavirenz, *DRV* darunavir, *LPV* lopinavir, *r* ritonavir, *RAL* raltegravir, *OD* once a day, *BID* twice a day

Patients considered their relationship to their physician as satisfactiory. They had “*a complete trust*” or “*absolute trust*” in them. Patients put forward medical support, empathy and psychological and social care: “*Coming to my hospital here is a support, there is always someone who will listen to me, wanting to know what I think”; “my doctor is always available, he made me overcome many fears regarding HIV”*.

ii) Perceptions of general vaccination

Half of the interviewees had a very favorable opinion and very strong positive feelings on vaccines, citing them as “*a benefit for humanity*”. Other patients did not understood the difference between mandatory and recommended vaccines. Only one patient had a negative view of vaccination and admitted that vaccines frightened her*.*

iii) Knowledge about vaccines

The majority of patients (12/20) admitted having limited knowledge about vaccines. They were unable to give a definition of therapeutic vaccination. Even when patients thought they had some knowledge, they were unable to explain the mode of action of therapeutic vaccine. One patient said “*Instead of taking medication, you get vaccinated”* or *“It’s several associated drugs. I’m not sure*”.

iv) Fear of injections

Only a few patients (6/20) expressed fear of injection. One stated it was a “*phobia*” for her.

v) Future characteristics of HIV therapeutic vaccines

The vaccination injection schedule was a key factor for most patients. The minimal acceptable interval between doses of HIV therapeutic vaccine was 6 months. A patient said “*Every year is OK, every month is more inconvenient”*; another one*: “the longer effective, the better it is”*. The future efficacy of therapeutic HIV vaccination on HIV viral load must be as effective as their ongoing ART: the minimal efficacy for undetectable viral load was between 80 and 100%.

vi) Expected benefits

Patients’ expected a therapeutic vaccine to reduce the daily burden of treatment with minimal side−effects; easing guilt feelings about transmitting HIV to others was also a major incentive.

A minority of patients (4/20) expected no change from the vaccine: “*the disease is still there”*, “*I am sick, I am sick, and nothing will change”*.

vii) Vaccination barriers

Several patients (8/20) feared the possible side−effects but were also concerned by the possibility of ineffectiveness *“If I’m not sure about the vaccine”.*

viii) Hope of a cure

The majority of patients (13/20) hoped for a cure, and believed that a therapeutic vaccine to cure HIV would be found in the future “*One day I think we will find a vaccine to cure the HIV virus*”.

## Discussion

To our knowledge, our study is the first to provide information from a western country on the acceptability by PLWHs for a future therapeutic HIV vaccine. Our study shows high indices of acceptance: the mean score for acceptability on the VAS was 8.4, and 92% of patients agreed to be vaccinated if a therapeutic vaccine becomes available.

HIV therapeutic acceptance depended on the expected characteristics of the vaccine, notably its duration of effectiveness: 44% of participants demanded the vaccine to be effective for life.

In other diseases, the situation is similar to HIV; there are several therapeutic vaccines in clinical development: HBV, HCV, HPV papilloma virus [34–36].

The only one therapeutic vaccine available since 2011 in the USA, is a prostate cancer therapeutic vaccine [37]. For this vaccine, there has not been publication about the acceptability. Similarly, there is no published acceptability analysis for vaccine candidates in other chronic viral diseases.

The only comparison we could make, is with efficient prophylactic vaccines, for which the acceptability is reported as rather low in France. That is why this study brings important focus.

The participants’ vaccine coverage was higher than those described in Mohseni et al. study [38]. Health-related Qol scores of the RAVVIH study patients were comparable to those reported in the VESPA 2 study [39].

Acceptance was not associated with socio-demographic, clinical (mode of contamination, duration of disease), or Qol and illness-perception parameters possibly because the expected benefits of future therapeutic HIV vaccines were so high that they overshadowed differences in patients’ characteristics. The acceptability was strongly correlated with the confidence in the treating physician. Even some patients that believed the doctors in general would not tell all the truth about vaccines, would have a strong trust in their physician and would agree to their advices.

Compared to those reported by patients suffering from diabetes or asthma [28], the Brief IPQ-R scores of our patients show that ‘identity’ was much less affected for PLWHs (2.1 ± 2.6) than for diabetic (4.6 ± 2.8) or asthmatic (4.5 ± 2.3) patients. However, PLWHs scored generally higher on ‘treatment control’ (9.1 ± 1.6 vs. 8.0 ± 2.3 [diabetes] and 7.9 ± 2.0 [asthma]) and ‘concern’ (8.1 ± 2.5 vs. 7.0 ± 3.1 [diabetes] and 4.6 ± 2.8 [asthma]) dimensions of the Brief IPQ-R. This suggests that treatment burden may be highly flustering to PLWHs currently on ART. Their scores on the ‘consequence’ dimension were comparable to those reported by patients suffering from diabetes.

In the field of future preventive HIV vaccines, Newman et al. [25] have already reported that acceptability is correlated with perceived effectiveness on transmission, possible side−effects, dose regimen and time intervals between doses [25].

However, while therapeutic vaccines are a rapidly growing new technology in medical science (several studies are ongoing on the efficacy of therapeutic vaccines, mainly for cancer [34]) however, studies on the representations and acceptability of these vaccines are lacking.

Only one study in Chinese PLWH (Dong et al. [27]) has adressed the willingness to participate in a therapeutic HIV-vaccine clinical trial and shown high rates of acceptance.

Our study has some limitations. The study has been proposed to all consecutive subjects, unhappily the number and the reasons for non-participation has not been recorded in a registry, but globally one quarter of patients did not accepted to participate, and mostly due to lack of time. This study is about the theoretical acceptability and it may be possible that in a real life decision situation some patients would decide differently. However the Patient’ self-Reported Outcome has showed its validity on different levels and is now considered as a valid endpoint by Health Authorities. The three clinics participating in this study may be more actively encouraging updating preventive vaccination coverage than other hospitals. Concerning the HBV vaccination 40% of patients were up to date versus 73% in our sample, concerning the Diphtheria-tetanus-inactivated poliovirus vaccination dTp: 56,2% of patients were up to date versus 72% in our sample. In fact, the uptake of immunization coverage of our study population was somewhat higher than the figures observed for other French hospitals [38].

The results from the RAVVIH study may not be directly applicable to other countries with health systems differing from France. The study does not assess some important dimensions of acceptance, such as cost as in France, ART is fully reimbursed.

Further, French guidelines for immunization for PLWHs recommend diphtheria, tetanus, poliomyelitis, pneumococcus, influenza and hepatitis B vaccines.

## Conclusion

This study explored the perceptions regarding a future therapeutic HIV vaccine in a representative sample of French PLWHs. PLWHs were receptive to the idea of receiving a therapeutic HIV vaccine. The vaccine’s characteristics and confidence in their treating clinician were acceptability’s key factors.

## Abbreviations

AIDS: Acquired immune deficiency syndrome
ART: Antiretroviral treatments
Brief IPQ R: Brief Illness Perception Questionnaire
GP: General practitioner
HIV: Human Immunodeficiency Virus
IDU: Intravenous Drug User
MSM: Men who have sex with men
PLWHA: People living with HIV Aids
PROQOL-HIV: Patient-Reported Outcomes Quality Of Life HIV instrument
Qol: Quality of live
RAVVIH: Representations and Acceptability of a HIV Therapeutic Vaccine
SAGE: Strategic Advisory Group of Experts
UNAIDS: United Nations Programme on HIV/AIDS
VAS: Visual analogue scale
WHO: World Health Organization
